# Identification of the m6A/m5C/m1A methylation modification genes in Alzheimer’s disease based on bioinformatic analysis

**DOI:** 10.18632/aging.206146

**Published:** 2024-10-31

**Authors:** Qifa Tan, Desheng Zhou, Yuan Guo, Haijun Chen, Peng Xie

**Affiliations:** 1 Ganzhou City Key Laboratory of Mental Health, The Third People’s Hospital of Ganzhou City, Ganzhou 341000, Jiangxi, China; 2 Guangzhou Medical University, Guangzhou 510182, Guangdong, China; 3 Department of Medical Genetics, Ganzhou Maternal and Child Health Hospital, Ganzhou 341000, China

**Keywords:** Alzheimer’s disease, cluster analysis, machine learning, immune characteristics, diagnostic model

## Abstract

Background: As a progressive neurodegenerative disease, the comprehensive understanding of the pathogenesis of Alzheimer’s disease (AD) is yet to be clarified. Modifications in RNA, including m6A/m5C/m1A, affect the onset and progression of many diseases. Consequently, this study focuses on the role of methylation modification in the pathogenesis of AD.

Materials and methods: Three AD-related datasets, namely GSE33000, GSE122063, and GSE44770, were acquired from GEO. Differential analysis of m6A/m5C/m1A regulator genes was conducted. Applying a consensus clustering approach, distinct subtypes within AD were identified as per the expression patterns of relevant differentially expressed genes. Machine learning models were constructed to identify five significant genes from the best model. The analysis of hub gene-based drug regulatory networks and ceRNA regulatory networks was conducted by Cytoscape.

Results: In comparison to non-AD patients, 24 genes were identified as dysregulated in AD patients, and these genes were associated with various immunological characteristics. Two distinct clusters were successfully identified through consensus clustering, with cluster 2 demonstrating higher immune characteristics compared to cluster 1. The performance of four machine learning models was determined by conducting a receiver operating characteristic (ROC) analysis. The analysis revealed that the SVM model achieved the highest AUC value of 0.947. Five genes (YTHDF1, METTL3, DNMT1, DNMT3A, ALKBH1) were selected as the predicted genes. Finally, a hub gene-based Gene-Drug regulatory network and a ceRNA regulatory network were successfully developed.

Conclusions: The findings offered fresh perspectives on the molecular patterns and immune mechanisms underlying AD, contributing valuable insights into our understanding of this complex neurodegenerative disorder.

## INTRODUCTION

Alzheimer’s disease (AD) is an intricate and progressive neurodegenerative disease predominantly impacting the elderly, influencing the activities of daily living and social functioning of affected individuals [1]. As of 2018, Alzheimer’s International approximated that approximately 50 million individuals globally are impacted by dementia, with projections indicating an increase to 152 million by 2050. Notably, two-thirds of these cases are expected to be concentrated in low- and middle-income countries [2, 3]. Numerous longitudinal studies have identified diverse risk and protective factors associated with AD, some of which can mitigate AD risk or delay its onset [4]. However, due to the clinical heterogeneity of AD and its complexity of pathological types, there is no effective way to prevent the occurrence of AD, and the disease still lacks strong effective treatment [5]. Further exploration into the pathogenesis of AD and the development of novel targets for its treatment is imperative. The utilisation of bioinformatics to develop multifactor predictive models holds the potential to offer fresh perspectives on individualized and precise treatment approaches for individuals with AD.

In eukaryotic transcriptome regulation, universal existence is noted for three prominent modifications, namely N1-methyladenosine (m1A), N6-methyladenosine (m6A), and 5-methylcytosine (m5C). This universal occurrence underscores the significance of these modifications in the intricate landscape of mRNA regulation within eukaryotic organisms. Advancements in epigenetic studies reveal mounting evidences linking gene methylation with the progression of AD. Several studies have noted differential methylation associated with AD [6–9]. The abundance of m6A is higher in the central nervous system than in other organs [10]. The m6A methylation modification genes not only promote self-renewal and proliferation of neural stem cells through multiple signaling pathways (such as JAK/STAT and PI3K/AKT) [11], but also regulate learning and memory by promoting synaptic transmission and transcription [12]. Dysregulation of m1A modifications in mitochondrial and cytosolic tRNA may induce the onset of Alzheimer’s disease by affecting protein synthesis [7], and similarly, mitochondrial m5C RNA methylation is essential for the dynamic regulation of mitochondrial translation rate [13]. Clustering subsets and risk models of m6A/m5C/m1A regulatory genes are associated with poor prognosis and immune microenvironment in a variety of cancers, promising to be a new tool for assessing patient outcomes [14, 15]. It is reasonable to suggest that m6A/m1A/m5C modifications are critically involved in AD progression. Nevertheless, the regulatory process of m6A/m1A/m5C in AD is currently unclear and requires further investigation. Bioinformatics was employed to collect data from the GEO website database, and a thorough analysis of the molecular mechanisms underlying AD pathogenesis and its immunological features was conducted.

## RESULTS

### Correlation analysis of methylation modification genes and immune properties in AD

**Figure 1:**
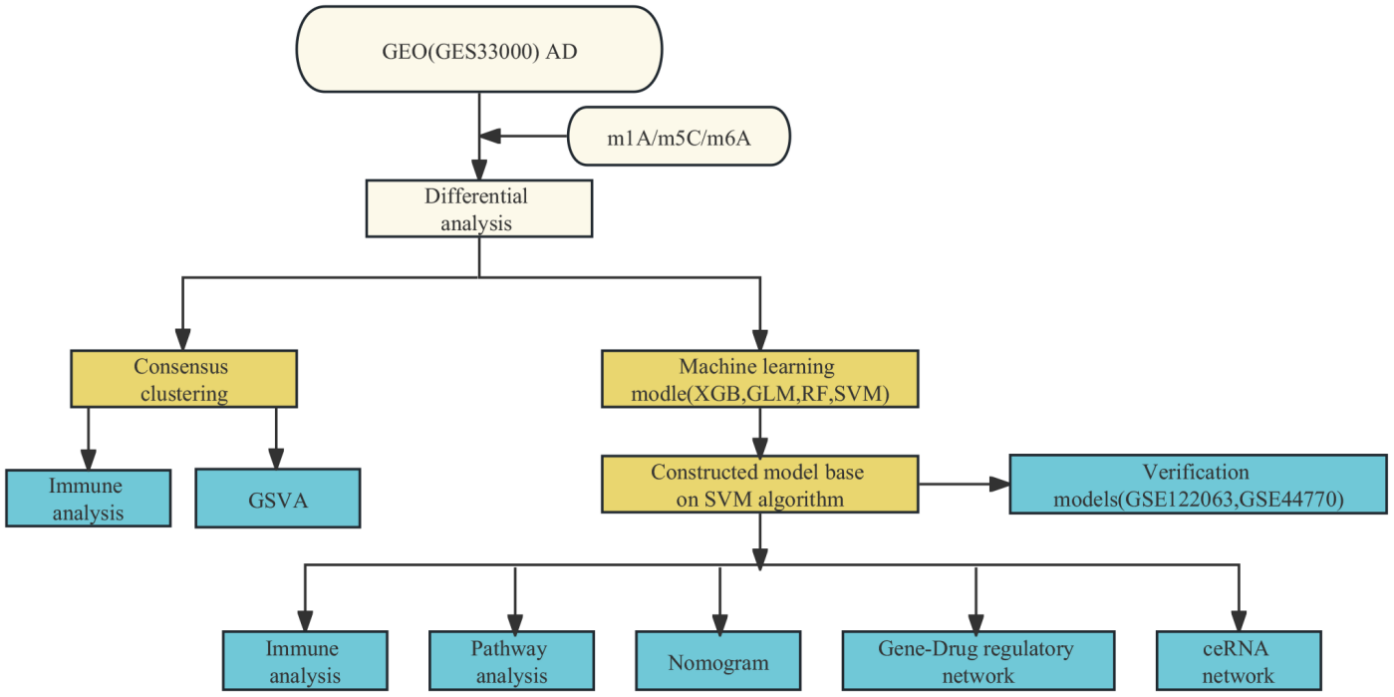
Study flow chart.

**Figure 2:**
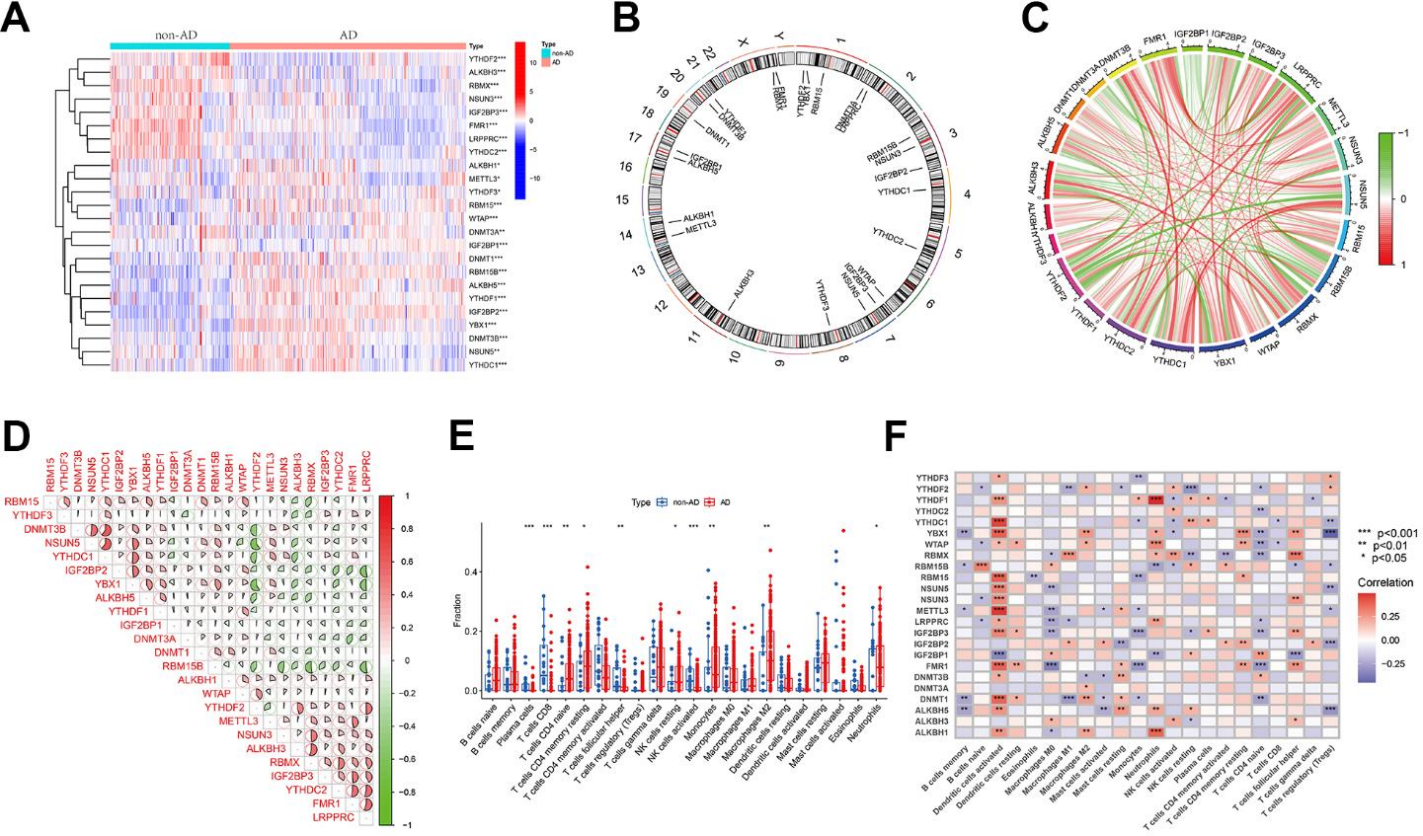
**Differential analysis of three regulators of methylation modification in Alzheimer’s disease.** (**A**) Heatmap illustrating the expression data of 24 regulators. (**B**) Chromosomal localization of 24 regulators. (**C**, **D**) Analysis of the correlation between the 24 differentially expressed regulatory factors, with red indicating positive association and green indicating negative association. The correlation coefficient is represented by the pie chart area. (**E**) Boxplots demonstrating variations in immune infiltration between AD and non-AD controls. *P < 0.05, **P < 0.01, ***P < 0.001. (**F**) Correlation analysis of 24 methylated differential genes with 22 immune cell types. *P < 0.05, **P < 0.01, ***P < 0.001.

Figure 1 depicts a comprehensive flow chart outlining the research route of this study. In the exploration of the biological significance of methylation regulators in AD, the GSE33000 dataset was employed for examining the expression profiles of 50 genes associated with three methylation modification gene sets. A total of 24 genes exhibited differential expression, with 15 (ALKBL1, ALKBL5, DNMT1, DNMT3B, IGF2BP1, IGF2BP2, METTL3, NSUN5, RBM15, RBM15B, WTAP, YBX1, YTHDC1, THDF 1 and THDF 3) exhibiting upregulation in individuals with AD, and 9 (ALKBL3, DNMT3A, FMR1, IGF2BP3, LRPPRC, NSUN3, RBMX, YTHDC2 and THDF 2) exhibiting downregulation compared to non-AD patients (Figure 2A). The locations of the 24 regulators on the chromosome were depicted in Figure 2B using the ‘RCircos’ package. Following this, correlation studies were conducted on DEGs to investigate the potential involvement of methylation regulators in AD development. Surprisingly, certain methylation regulators, such as YTHDC1 and DNMT3B, along with YTHDC1 and NSUN 5, showed strong synergies. Meanwhile, YTHDF2 and NSUN5, along with YTHDF2 and YBX 1, showed a competing relationship (Figure 2C, 2D).

In the CIBERSORT algorithm, an analysis of immune infiltration was performed to assess variations in the percentages of 22 infiltrated immune cell types between the AD and non-AD groups. As shown in Figure 2E, the percentages of activated NK cells, CD8^+^ T cells, and follicular helper T cells were reduced in AD samples relative to healthy samples, whereas the opposite was true for resting NK cells, naive CD4^+^ T cells, monocytes, resting memory CD4^+^ T cells, M2 macrophages, and neutrophils. In the subsequent analysis, the link between 24 DEGs and immune cell infiltration was examined. The findings revealed that multiple genes exhibited negative correlations with memory B cells, M0 macrophages (excluding RBM15B, ALKBH3, and IGF2BP1), naive CD4^+^ T cells (excluding IGF2BP1 and DNMT3B), and regulatory T cells (excluding YTHDF2 and YTHDF3). Conversely, positive associations were connected with activated DC cells (excluding IGF2BP1), neutrophils (excluding RBM15B and IGF2BP1), resting memory CD4^+^ T cells, and follicular helper T cells (excluding RBM15B and IGF2BP1) (Figure 2F). The findings indicate the potential involvement of methylation-regulating genes in AD development by affecting the level of immune cell infiltration.

### Identification of methylation-modifying gene clusters and differential analysis of immune features in AD

**Figure 3:**
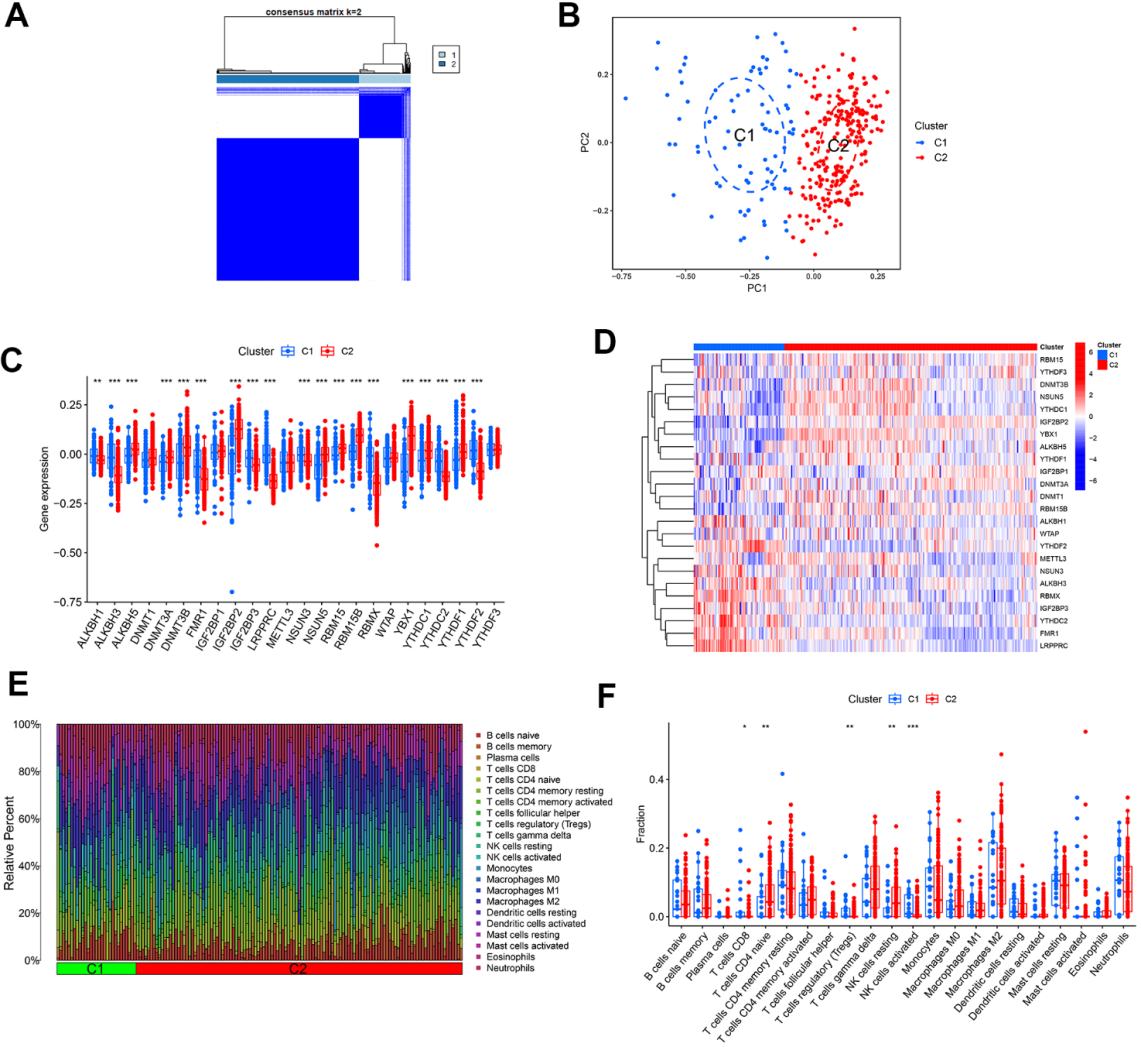
**Determination of molecular clusters related to m1A, m5C, and m6A in AD.** (**A**) Consensus clustering matrix when k = 2. (**B**) PCA analysis. (**C**) Boxplots illustrating the expression of 24 DEGs between two clusters. (**D**) Heatmap displaying a differential expression of 24 DEGs between the two clusters. (**E**) Relative percentages of 22 infiltrated immune cells between two clusters. *P<0.05, **P<0.01, ***P<0.001. (**F**) Boxplots depicting variations in immune infiltration between two clusters. * *P* < 0.05, ** *P* < 0.01, ***P < 0.001.

To investigate the impact of methylation modification regulators in AD, a consensus cluster analysis was performed to examine potential novel molecular subtypes among individuals with AD. So, 310 AD samples were classified into 2 clusters according to the expression profiles of 24 regulatory factors, setting k = 2 yielded the most consistent cluster values (Figure 3A), and the PCA revealed different transcription rates between the two clusters (Figure 3B). Moreover, cluster 1 showed elevated expression levels of ALKBH1, ALKBH3, FMR1, IGF2BP3, LRPPRC, NSUN3, RBMX, YTHDC2, and YTHDF2 genes, whereas cluster 2 exhibited elevated expression levels of ALKBH5, DNMT3A, DNMT3B, IGF2BP2, NSUN5, RBM15, RBM15B, YBX1, YTHDC1, and YTHDF1 genes (Figure 3C, 3D). Immune infiltration analysis further demonstrated variations in the immune microenvironment between the two clusters (Figure 3E). Cluster 1 displayed a higher prevalence of CD8^+^ T cells, regulatory T cells, and activated NK cells, whereas cluster 2 displayed a higher prevalence of naive CD4^+^ T cells and resting NK cells (Figure 3F).

### GSVA functional analysis

**Figure 4:**
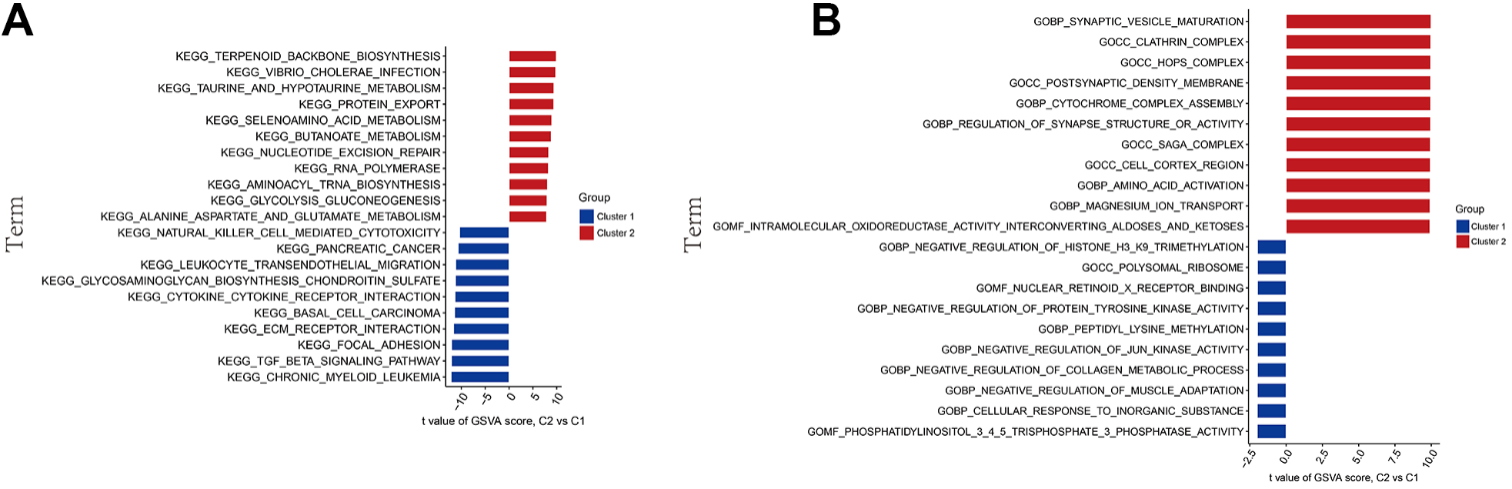
**GSVA biological functional analysis.** (**A**) The KEGG pathway analysis. (**B**) The GO function analysis.

To delve deeper into the functional differences between the two groups of methylation modification gene clusters, GSVA was employed. The findings demonstrated that cluster 1 exhibited heightened natural killer cell-mediated cytotoxicity, cytokine-cytokine receptor interaction and TGF-β signaling pathway. Conversely, cluster 2 demonstrated activation of pathways related to terpenoid skeleton biosynthesis, vibrio cholerae infection, and metabolism (Figure 4A). Moreover, functional enrichment analysis highlighted that cluster 1 was related to protein tyrosine kinase activity and negative regulation of JUN kinase, and cluster 2 was associated with the mature development of synapses, regulation of cytochrome complex assembly, and amino acid activation (Figure 4B).

### Development of machine-learning models

**Figure 5:**
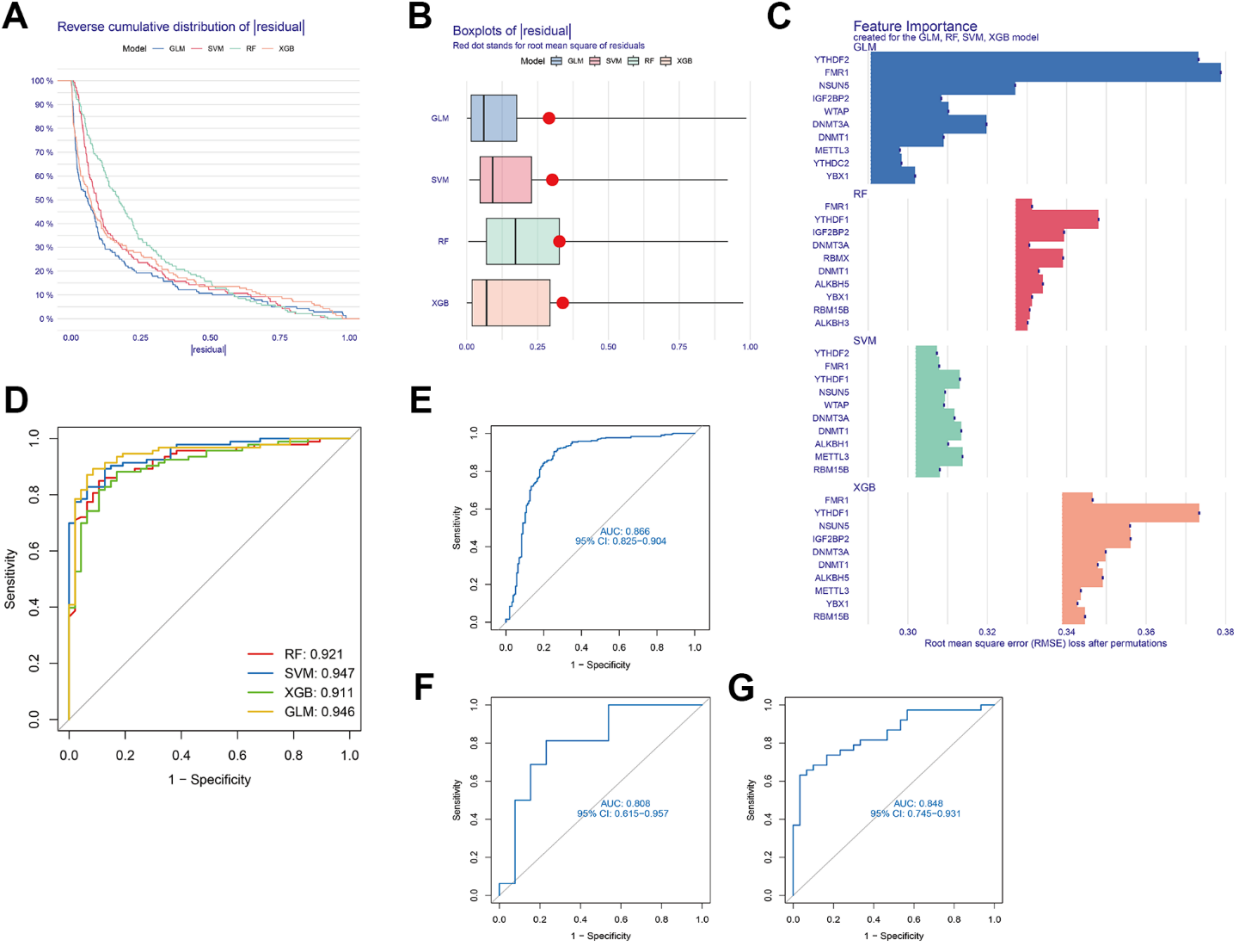
**Development of a suitable diagnostic model through four machine learning and validation models.** (**A**) Distribution of cumulative residuals for each machine learning model. (**B**) Boxplots illustrating the residuals of all machine learning models. (**C**) The salient characteristics of the RF, SVM, GLM, and XGB machine learning models. (**D**) Four machine learning models tested utilising a fivefold cross-validation procedure, with results examined utilising the ROC curve. (**E**) ROC analysis of the 5-gene-based SVM model in GSE33000 dataset. (**F**) ROC analysis of the 5-gene-based SVM model in GSE122063 dataset. (**G**) ROC analysis of the 5-gene-based SVM model in GSE44770 dataset.

For exploring the link between methylation regulators and AD subtypes, four machine learning models—GLM, RF, SVM, and XGB—were developed using the 24 DEGs in the AD training set. The objective was to identify genes specific with high diagnostic value. The interpretation of these four models and visualisation of the residual distribution for every model in the test set were accomplished by the R “DALEX” package. Notably, the GLM and SVM models exhibited lower residuals (Figure 5A, 5B). Subsequently, ten key genes were identified from the four modules, ranked based on root mean square error (RMSE) (Figure 5C). Additionally, the diagnostic efficacy of the four models was determined through the ROC curve. Notably, the SVM model demonstrated the highest diagnostic power (AUC = 0.947) (Figure 5D). Collectively, these findings highlight the superiority of the SVM model in distinguishing between patient groupings. Following the execution of the SVM model, the top five variables (YTHDF1, METTL3, DNMT 1, DNMT3A, ALKBH1) were chosen as predictor genes. The ROC analysis of the five genes based on the SVM model in the 3 GEO datasets (GSE33000, GSE122063, and GSE44770) was illustrated in Figure 5E–5G. Notably, the AUC values for all three GEO datasets exceeded 0.8, signifying that the model constructed by SVM possessed a high diagnostic value.

### Correlation analysis between hub genes and immune characteristics

**Figure 6:**
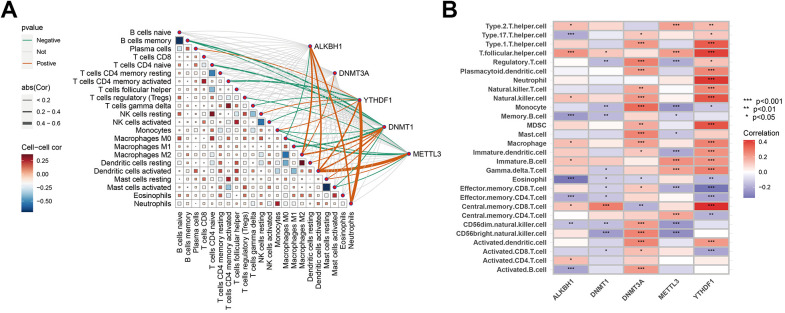
**Correlation analysis between hub genes and immune characteristics.** (**A**) Correlation between hub genes and immune cells shown by CIBERSORT analysis. (**B**) Association between hub genes and immune cells depicted by ssGSEA analysis. The colour spectrum, ranging from red to purple, illustrates the transition from positive to negative associations, respectively. A high number of asterisks and darker-coloured modules depict stronger associations. *P < 0.05; **P < 0.01; ****P < 0.001.

In exploring the association between hub genes and immune cells, various algorithms were employed for analysis. By using the CIBERSORT algorithm, it was observed that Macrophages M2 exhibited positive correlations with ALKBH1, DNMT1, and DNMT3A, whereas activated DC exhibited positive correlations with ALKBH1, DNMT1, METTL3, and YTHDF1. Neutrophils were positively correlated with ALKBH1 and YTHDF1. In addition, DNMT3A and YTHDF1 were significantly negatively correlated with other immune cells, encompassing monocytes, memory B cells, M0 Macrophages, M1 Macrophages, etc. Moreover, through ssGSEA, it was found that DNMT3A and YTHDF1 exhibited significant positive associations with the majority of immune cells, whereas DNMT1 and METTL3 exhibited negative correlations with a broader range of immune cells (Figure 6A, 6B). These results implied that the occurrence of AD may be associated with alterations in the immune microenvironment.

### Strong association of hub genes with AD-related pathways

**Figure 7:**
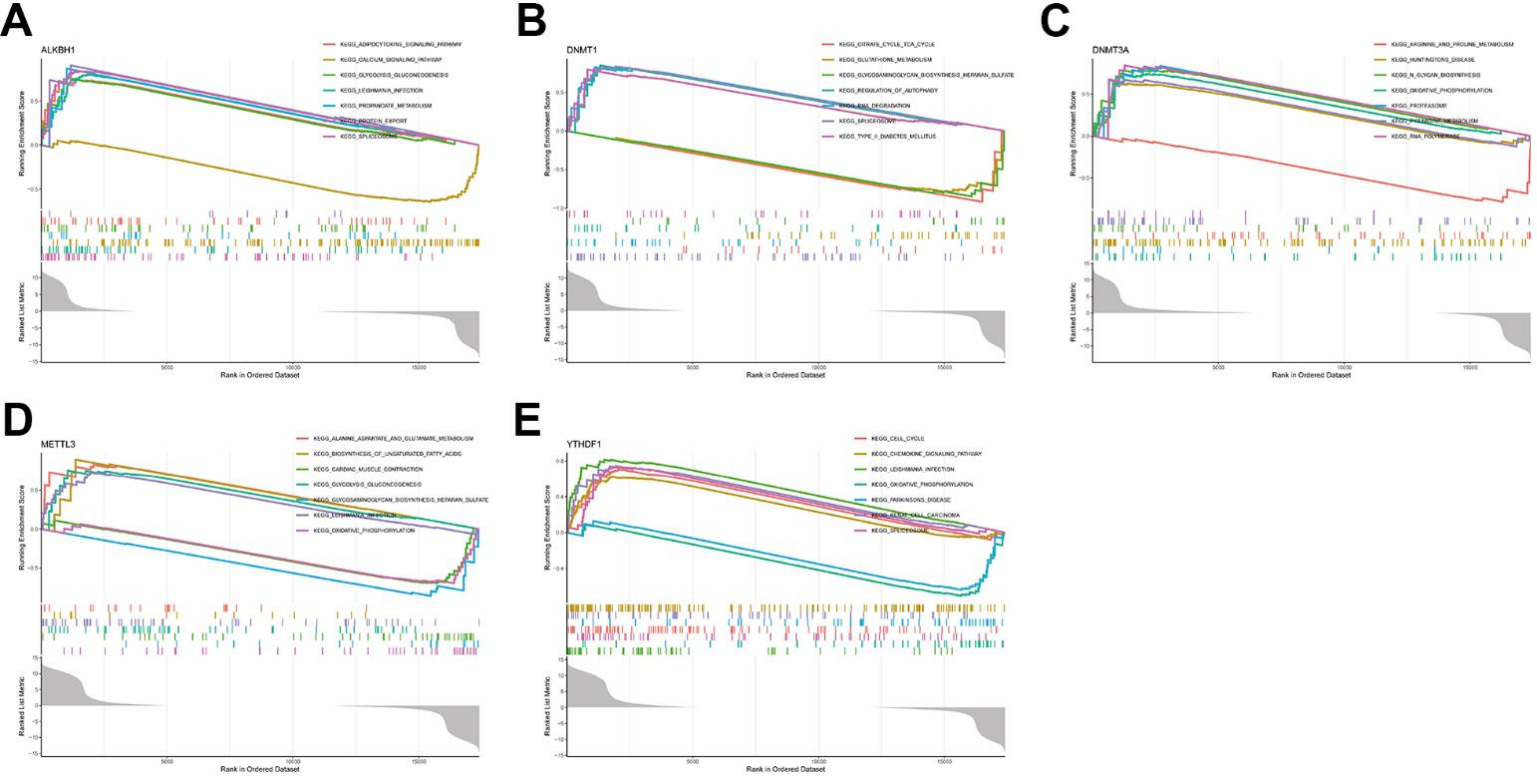
**Single-gene GSEA-KEGG pathway analysis.** (**A**) ALKBH1. (**B**) DNMT1. (**C**) DNMT3A. (**D**) METTL3. (**E**) YTHDF1.

For an in-depth understanding of the involvement of hub genes in AD development, a single-gene GSEA was conducted. The findings reveal the top 7 pathways for each gene enrichment (Figure 7A–7E). The analysis showed that ALKBH1, DNMT1, DNMT3A, and METTL3 were involved in the metabolism-related pathway. In addition, ALKBH1, DNMT1, and YTHDF1 genes were involved in spliceosome signalling, while DNMT3A, METTL3, and YTHDF1 were associated with oxidative phosphorylation. Moreover, the function of the YTHDF1 gene also pointed to the chemokine signalling pathway.

**Figure 8:**
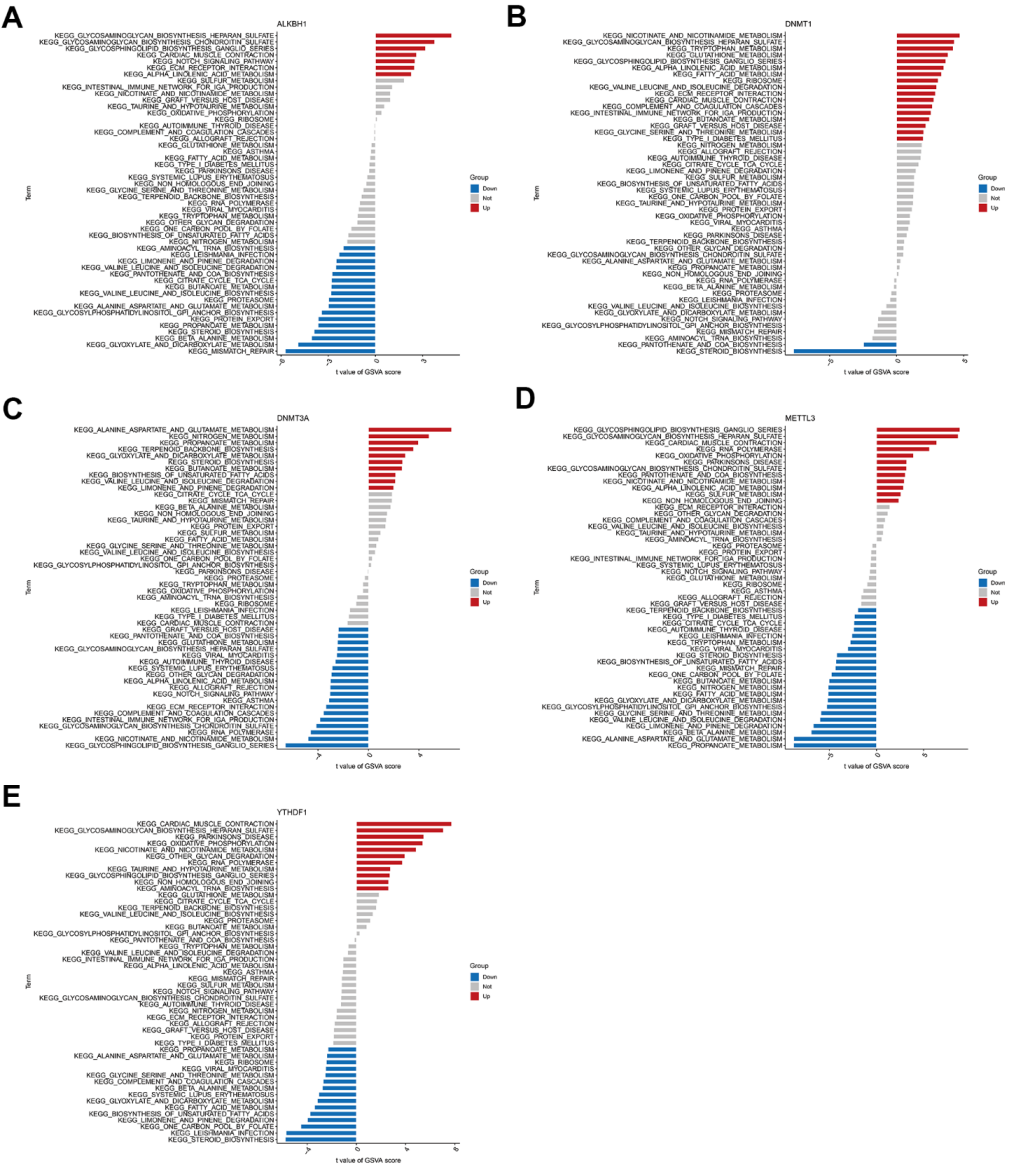
**High and low expression groups per the expression levels of each marker gene combined with GSVA.** (**A**) ALKBH1. (**B**) DNMT1. (**C**) DNMT3A. (**D**) METTL3. (**E**) YTHDF1.

Further enrichment analysis of hub genes was conducted using GSVA (Figure 8A–8E). This analysis predicted and revealed variations in the activated pathways between the high- and low-expression groups of these genes. The results showed that the upregulation of genes ALKBH1, DNMT1, and METTL3 and the downregulation of DNMT3A could jointly activate the Alpha-Linolenic acid metabolism pathway. Activation of the Glycosaminoglycan biosynthesis heparan sulfate pathway was associated with the up-regulation of genes ALKBH1, DNMT1, METTL3, and YTHDF1 expression, as well as down-regulation of DNMT3A expression. Reduced expression levels of ALKBH1, METTL3, and YTHDF1 and elevated DNMT1 and DNMT3A expression levels affect the activation of several amino acid metabolic pathways. Cardiac contraction was related to the upregulation of ALKBH1, DNMT1, METTL3, and YTHDF1 expression. Moreover, reduced expression levels of DNMT3A, METTL3, and YTHDF1 were associated with immune diseases or immune signalling pathways. These results suggested that alterations in the immune microenvironment of individuals with AD may be linked to these five hub genes.

### Development of nomogram

**Figure 9:**
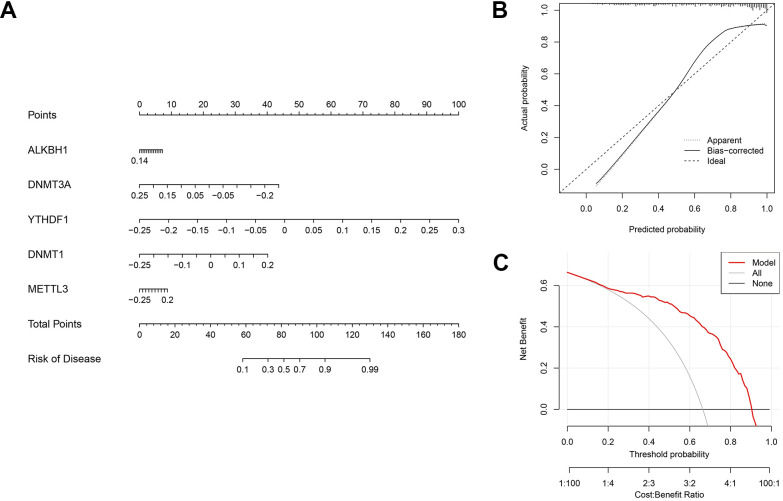
**Validation of the five-gene based on the SVM model.** (**A**) Development of a nomogram utilising the 5-gene based on the SVM model to predict the risk of AD patients. (**B**) Assessment of the prognostic efficacy of the nomogram model through a calibration curve. (**C**) Utilisation of discriminant analysis for evaluating the sensitivity of the nomogram to change.

A nomogram was developed to evaluate the prognostic capability of the SVM model across diverse AD datasets (Figure 9A). Within the nomogram, each gene corresponded to a specific scoring criterion, and the cumulative scoring of all genes then predicted the risk of AD progression. The calibration curve of the nomogram exhibited a robust predictive performance (Figure 9B). Furthermore, the decision curve (Figure 9C) analysis revealed that the nomogram provided higher clinical benefit for patients through a comprehensive score of five methylation-related genes.

### Prediction of marker gene-targeted drugs

**Figure 10:**
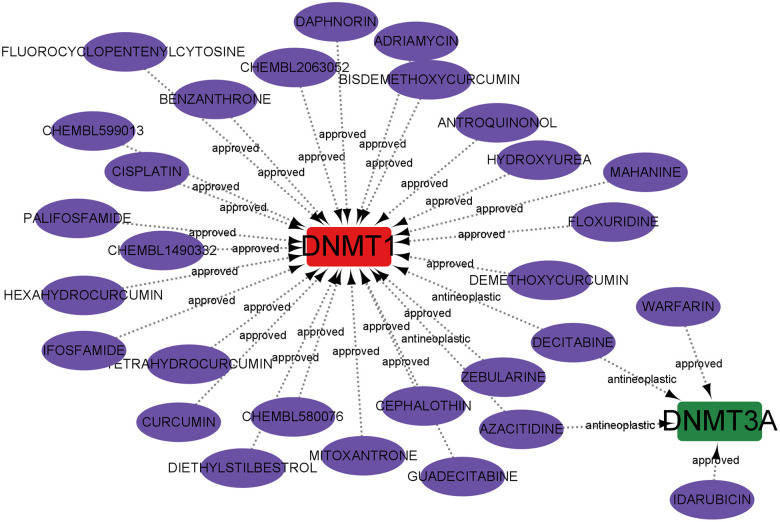
Prediction of marker gene-targeted drugs.

The Drug-Gene Interaction Database was utilised for predicting potential drugs targeting the hub genes, and the relationships between genes and drugs were examined. By inputting the five core genes into the DGIdb website, only two core genes related to predictive drug information were retrieved and visualised by using Cytoscape software. Overall, 29 drugs acting on the hub genes were identified. Among them, four drugs targeted DNMT3A, and 27 drugs targeted DNMT1. Notably, Decitabine and azacytidine may exhibit therapeutic efficacy in AD by targeting the expression of DNMT1 and DNMT3A. Unfortunately, drug targets for ALKBH1, METTL3, and YTHDF1 were not predicted (Figure 10).

### Construction of the ceRNA network

**Figure 11:**
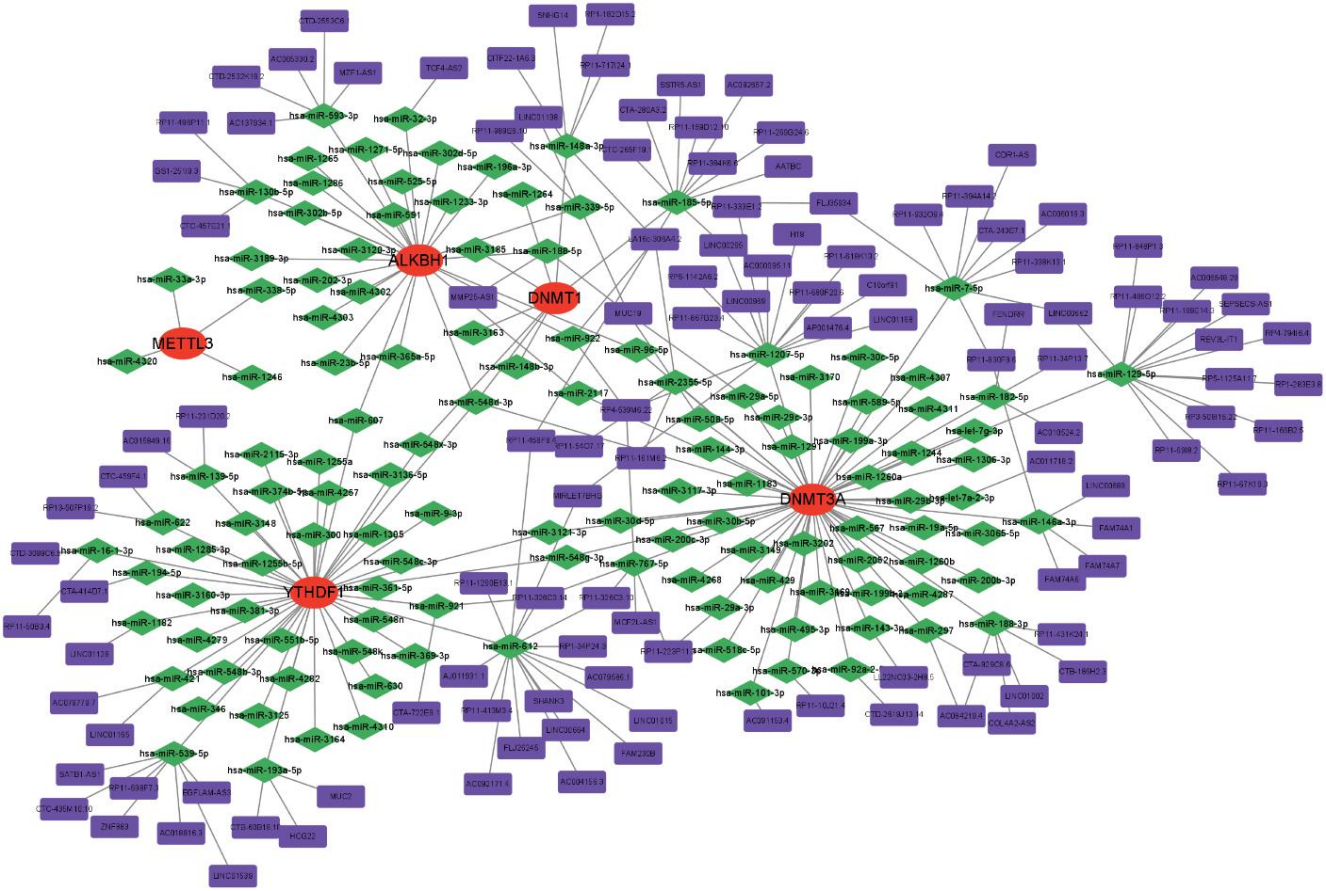
Construction of a network of ceRNA based on hub genes.

Numerous studies have substantiated the involvement of ceRNA regulatory networks in the biology and pathophysiology of multiple diseases. To investigate if central genes exhibit identical regulatory relationships in AD, a ceRNA network based on hub genes was established. TargetScan [16], miRNet [17], and miRWalk [18] were utilised to identify the five targeted hub gene miRNAs shared by these three databases. Subsequently, spongeScan data was employed to identify the lncRNAs interacting with these miRNAs. Ultimately, the ceRNA network was visualised by Cytoscape, comprising a total of 252 nodes, which included 5 mRNAs, 127 miRNAs, and 120 lncRNAs (Figure 11).

## DISCUSSION

AD, characterised by its heterogeneity and intricate pathobiology, presents a notable challenge due to the absence of effective disease-modifying treatments. Unfortunately, the outcomes of numerous phase 3 clinical trials have been disappointing, failing to reveal substantial benefits [19]. The FDA has approved only a specific set of drugs, such as acetylcholinesterase inhibitors and N-methyl-D-aspartate antagonists, for the explicit purpose of treating cognitive impairment in individuals with AD [19]. Beyond pharmacotherapy, interventions that target multiple risk factors simultaneously in various domains may prove effective in dementia prevention strategies. Nevertheless, it is acknowledged that multi-domain interventions can be demanding and may not be universally accepted [20]. Adherence exhibited a decline with the escalating complexity and intensity of the intervention [21]. Considering the heterogeneity in disease symptoms, physical conditions, and patient preferences, a ‘one-size-fits-all’ intervention is not applicable. Consequently, this study aimed to enhance the comprehension of how methylation-modification-related genes operate within the AD phenotype and immune microenvironment. This understanding is crucial for facilitating the diagnosis and promoting individualized treatment approaches for AD.

Methylation modifications play regulatory roles in diverse cellular processes by influencing the transcription, maturation, localization, function, and metabolism of various RNA classes [22]. For example, elevated m6A methylation levels in CXCL2 and IFNG mRNA have been associated with decreased mRNA stability and translation, thereby inhibiting CD4^+^ T cell responses [23]. m1A levels have been negatively correlated with CD8^+^ T effector cell proliferation in colon cancer [24]. Additionally, m5C-methylation of IL17A mRNA has been found to enhance its translation in T lymphocytes [25]. A growing number of studies are exploring machine learning techniques with novel biomarkers as promising methods for predicting AD [26, 27].

Given that the majority of prior research on AD was based on a single gene cluster, this research aimed to enhance the comprehensiveness of the assessment. It identified 50 gene expression profiles related to m6A, m1A, and m5C. The initial comparison involved scrutinising the expression data of m6A/m1A/m5C regulators in brain tissue from both healthy participants and individuals with AD. This detailed analysis revealed 24 DEGs. Subsequent correlation analysis provided insights into the intricate relationships, highlighting instances of strong synergistic or antagonistic effects among several modulators. Research has documented the involvement of the immune microenvironment in individuals with AD, with the dysregulation of the immune response considered to underlie the onset and progression of AD [28]. CIBERSORT analysis revealed variations in immune infiltration between AD and non-AD patients. In AD patients, elevated levels of infiltration were observed in resting NK cells, naive CD4^+^ T cells, resting memory CD4^+^ T cells, M2 macrophages, monocytes, and neutrophils. This pattern is almost consistent with findings from previous studies [29, 30]. Multiple DEGs also showed correlations with the immune-infiltrating cells. The genes ALKBH1 and DNMT3A exhibited significant and positive correlations with M2 macrophages. DNMT1 showed a notable negative association with naive CD4^+^ T cells. METTL3 displayed a positive link to activated DCs and a negative link to regulatory T cells. YTHDF1 showed a positive association with neutrophils. This evidence indicates the involvement of hub genes in the regulation of the immune microenvironment in individuals with AD.

In a more in-depth analysis of the DEGs, the samples from individuals in the AD group were classified into Cluster 1 and 2 types through cluster analysis. The GSVA results indicated that cluster 2 was more active in taurine and glutamate-associated metabolic pathways. Additionally, it was associated with cellular cortical regions and regulation of synaptic vesicle maturation, postsynaptic density membrane, as well as synaptic structure or activity. This suggests that cluster 2 has more pathways that contribute to enhancing learning and memory functions, potentially delaying AD progression.

Finally, based on the multiple machine learning algorithm, four diagnostic models were constructed by using DEGs. Among these models, the SVM model displayed the highest predictive efficacy in the training cohort. Subsequently, five variables (YTHDF1, METTL3, DNMT1, DNMT3A, and ALKBH1) were selected as the predicted genes. Combining with the calibration curves of the nomogram, a robust concordance was observed between the predicted and actual observed values. Pathway-related single-gene GSEA further confirmed the involvement of hub genes in the immune microenvironment. Simultaneously, a gene-drug regulatory network was predicted and constructed as per the hub genes, offering a theoretical foundation for the development of targeted immunotherapy for AD. Taking into account the potential regulatory roles of miRNAs and lncRNAs on mRNAs, a ceRNA network was established by using hub genes, enhancing our comprehension of its molecular regulatory mechanism. In addition, drugs may also influence hub gene expression through regulatory RNA interactions, and if there are researchers engaged in in-depth drug development, these two charts deserve reference.

Yin’s study identified that the deficiency of METTL3 in monocyte-derived macrophages impairs YTHDF1-mediated DNMT3A translation, subsequently improving cognitive function in an amyloid beta (Aβ)-induced AD mouse model [31]. Mutations in DNMT1 caused hereditary sensory neuropathy with dementia and hearing loss [32]. ALKBH1 played a role in neural development by modifying the methylation status of histone H2A [33]. These research findings partially validated the effectiveness of our constructed diagnostic model. However, further validation is needed through additional clinical data and experimental studies.

## CONCLUSIONS

This study elucidated the involvement of the m6A/m1A/m5C regulator in AD development and its link to immune cell infiltration. The selected 5-gene-based SVM model emerged as the optimal machine learning tool for precisely assessing the risk of distinct AD subtypes. Additionally, the construction of gene-drug regulatory and ceRNA networks provided deeper insights into the molecular regulatory mechanisms of AD. It is crucial to note that all conclusions drawn are based on the analysis of publicly available data, emphasizing the necessity for further validation through additional clinical data collection and experimental studies.

## MATERIALS AND METHODS

### Data collection

Data collection involved the acquisition of three datasets pertaining to AD from the GEO database (https://www.ncbi.nlm.nih.gov/geo/), including GSE33000, GSE122063, and GSE44770. The gene expression data derived from human prefrontal cortex brain tissue were utilised as a pivotal criterion in this study. Within the GSE33000 database (platform GPL4372), 157 samples from healthy individuals and 310 samples from individuals with AD constituted the training group. Two other databases were utilised as test groups: the GSE122063 database (platform GPL16791), encompassing 44 AD samples and 56 healthy samples, and the GSE44770 database (platform GPL4372), comprising 129 AD samples and 101 healthy samples (Table 1). Normalization and processing of gene expression profiles of the three datasets were conducted utilising a “Perl” script and the R “limma” package. Following this, 50 gene expression profiles were identified for three methylation-modified gene (MMG) sets of m1A, m5C, and m6A, with 24 differentially expressed genes (DEGs).

**Table 1:** Alzheimer’s disease (AD)-related microarray datasets.

**Location**	**Dataset**	**Platform**	**Number**
Brain	GSE33000	GPL4372	157 control vs 310 AD
Brain	GSE122036	GPL16791	44 control vs 56 AD
Brain	GSE44770	GPL4372	101 control vs 129 AD

### Immune infiltration analysis

For the estimation of the relative abundance of 22 different immune cell types in AD samples, the CIBERSORT algorithm was applied [34]. This algorithm provided an insightful analysis of the composition of immune cells within the samples, enhancing our understanding of the immune microenvironment. To determine the link between MMGs and the AD immune microenvironment, the single sample gene set enrichment analysis (ssGSEA) method was applied [35] to analyse the correlations between hub genes and 28 different immune cell infiltrations. The analysis was conducted with a significance threshold set at *P*<0.05. The outcomes were expressed by utilising R packages “reshape2” and “ggpubr”.

### Consensus clustering for individuals with AD

Based on the expression of MMG DEGs in AD, the classification of AD samples into specific subtypes related to MMG was achieved by using the R “Consensus Cluster Plus” package. The maximum cluster number, k = 9 was selected, and the optimal cluster number was evaluated based on the consensus matrix (CM) and the cumulative distribution function (CDF). This method ensured a robust and accurate identification of distinct clusters within the dataset. Following these steps, the assessment of the distribution across clusters associated with MMG was performed utilising Principal Component Analysis (PCA).

### GSVA

This research employed gene set variation analysis (GSVA), an advanced methodology for pathway-level differential analysis, to explore variations in biological activities across MMG clusters. An enrichment study was conducted utilising the R “GSVA”. GSVA gene sets were acquired from the “curated gene sets” and “ontology gene sets” modules within the Molecular Signatures Database (MSigDB) (http://software.broadinstitute.org/gsea/msigdb/).

### GSEA

To scrutinise the variation in functional pathways and biological processes among hub genes in AD, gene set enrichment analysis (GSEA) was executed. Gene sets linked to diverse hallmarks were acquired from the MSigDB.

### Establishment of machine-learning models and development of a nomogram

Order to construct an AD diagnosis model, random forest model (RF) [36], support vector machine model (SVM) [37], eXtreme Gradient Boosting (XGB) [38] and generalized linear model (GLM) [39] machine learning models were constructed by repeatedcv, svmRadial, xgbDART and GLM methods, and R “caret”, “dalx”, “randomForest”, “kernlab” and “GLM” packages were used. The ROC curve was plotted using the R “pROC” package to assess the reliability of the model, identifying the top five predictive genes associated with AD through the optimal machine learning model. Subsequently, the diagnostic effect of the model was verified by ROC curve analysis on the GSE44770 and GSE122063 datasets. The risk prediction of individuals with AD was facilitated utilising a nomogram developed from the five essential genes determined by the SVM model. For a thorough assessment of the prognostic significance of the nomogram, decision curve analysis and the examination of a calibration curve were performed. These analyses served to verify the effectiveness of the predictive model through a comprehensive evaluation.

### Development of gene-drug regulatory networks

The Drug-Gene Interaction Database, an online repository sourced from DGIdb (https://www.dgidb.org/), was used in this study. The hub genes list was submitted to the database to retrieve essential details such as interaction scores, the nature of the interaction, and comprehensive data about various drugs for the gene. Using this acquired information, a gene-drug regulatory network was constructed to identify possible drug targets. The visualisation of these intricate gene-drug regulatory networks was accomplished by using the Cytoscape software.

### Development of the competitive endogenous RNA regulatory network

RNAs have the capacity to regulate each other through competition for binding to a common miRNA, a regulatory mode referred to as competitive endogenous RNA (ceRNA). The identified ceRNAs encompass both protein-coding mRNAs and non-coding RNAs, with the latter including lncRNAs and circRNAs. In this research, a ceRNA regulatory network was constructed involving interactions between mRNA, miRNA, and lncRNA with detailed methods outlined in ref [40]. Visualisation of these ceRNA regulatory networks was achieved by using Cytoscape.

### Statistical analysis

Statistical analysis was performed by using R software (V 4.1.1), with data processing executed through Perl and R “limma” package. For continuous variables, normality was assessed, and either the Student’s t-test or Wilcoxon rank-sum test was employed for analysis. All *P*-values of statistical data were derived from two-sided tests. *P*<0.05 was deemed as a statistically significant value.

### Data availability statement

The raw data of this study are derived from the GEO database (https://www.ncbi.nlm.nih.gov/geo/).
